# Immune Correlates of Natural HIV Elite Control and Simultaneous HCV Clearance—Supercontrollers

**DOI:** 10.3389/fimmu.2018.02897

**Published:** 2018-12-10

**Authors:** Beatriz Dominguez-Molina, Sara Ferrando-Martinez, Laura Tarancon-Diez, Jose Hernandez-Quero, Miguel Genebat, Francisco Vidal, Mª Angeles Muñoz-Fernandez, Manuel Leal, Richard Koup, Ezequiel Ruiz-Mateos

**Affiliations:** ^1^Clinic Unit of Infectious Diseases, Microbiology and Preventive Medicine, Institute of Biomedicine of Seville, IBiS, Virgen del Rocío University Hospital, Seville, Spain; ^2^Laboratory of Immunovirology, Institute of Biomedicine of Seville, IBiS, Virgen del Rocío University Hospital/CSIC/University of Seville, Seville, Spain; ^3^Immunology Laboratory, Vaccine Research Center, National Institute of Allergy and Infectious Diseases, National Institutes of Health, Bethesda, MD, United States; ^4^Hospital San Cecilio, Internal Medicine, Granada, Spain; ^5^Hospital Universitari Joan XXIII, IISPV, Universitat Rovira i Virgili, Tarragona, Spain; ^6^Sección Inmunología, Laboratory InmunoBiología Molecular, Hospital General Universitario “Gregorio Marañón”, Madrid, Spain; ^7^Instituto de Investigación Sanitaria del Gregorio Marañón, Madrid, Spain; ^8^Servicio de Medicina Interna, Hospital Viamed, Santa Ángela de la Cruz, Seville, Spain

**Keywords:** HIV, controllers, HCV, spontaneous clearance, T-cells, pDCs, NK, supercontrollers

## Abstract

HIV-elite controllers are a minority group of HIV-infected patients with the ability to maintain undetectable HIV viremia for long time periods without antiretroviral treatment. A small group of HIV-controllers are also able to spontaneously clear the hepatitis C virus (HCV) whom we can refer to as “supercontrollers.” There are no studies that explore immune correlates looking for the mechanisms implicated in this extraordinary phenomenon. Herein, we have analyzed HCV- and HIV-specific T-cell responses, as well as T, dendritic and NK cell phenotypes. The higher HCV-specific CD4 T-cell polyfunctionality, together with a low activation and exhaustion T-cell phenotype was found in supercontrollers. In addition, the frequency of CD8 CD161^high^ T-cells was related with HIV- and HCV-specific T-cells polyfunctionality. Interesting features regarding NK and plasmacytoid dendritic cells (pDCs) were found. The study of the supercontroller's immune response, subjects that spontaneously controls both chronic viral infections, could provide further insights into virus-specific responses needed to develop immunotherapeutic strategies in the setting of HIV cure or HCV vaccination.

## Introduction

HIV-controllers are a rare group of HIV-infected patients who can maintain HIV viremia at low levels in the absence of antiretroviral treatment (1, 2). Although several efforts are being made to elucidate mechanisms responsible of this phenomenon, multiple factors seem to be involved (3, 4). Interestingly, the extraordinary capacity of these subjects allows them to show differential characteristic regarding hepatitis C virus (HCV) coinfection. We have previously communicated that Caucasian HIV-controllers exhibited lower HCV viral loads (VL) than non-controllers, and a different HCV genotype distribution (5). Furthermore, Sajadi et al. reported a high rate of spontaneous clearance of HCV among HIV-controllers (6). We referred as supercontrollers those patients that simultaneously, and spontaneously, control HIV and clear HCV. The existence of these patients suggests the idea of some common or additive mechanisms concur in the control of both viruses.

Virus-specific T-cell response has been extensively studied and a higher virus-specific T-cell response and polyfunctionality has been unequivocally related with HIV and HCV control (7, 8). Besides, innate immunity has been also related with natural viral control. Higher NK frequency and anti-viral activity has been found in HIV-controllers (9, 10) and a high differentiated NK phenotype (defined by CD57 and HLA-C-binding killer cell immunoglobulin-like receptor (KIR) expression) has been reported in HCV spontaneous clearers (11, 12). Also, myeloid dendritic cells (mDCs) and plasmacytoid dendritic cells (pDCs) frequency, phenotype and antigen-presenting capacity have been related with HIV control (13–15) and HCV clearance (16, 17). However, there are no studies focused on patients which spontaneously control both viruses. For this purpose, we have selected the extraordinary group of supercontrollers to perform deep immunophenotyping of T-cells, NK and dendritic cells and to analyze HCV- and HIV-specific T-cell responses in order to find shared mechanisms of control, and to know whether supercontrollers have immunological characteristics that enable them to control more than one virus infection.

## Materials and Methods

### Samples and Patients

Peripheral blood samples from 50 HIV and HCV co-infected patients were collected from three Spanish Hospitals: Virgen del Rocio University Hospital (Seville), San Cecilio University Hospital (Granada) and Joan XXIII University Hospital (Tarragona). Of those, eight were HCV spontaneous clearers and HIV-elite controllers (supercontrollers, SC), 14 non-spontaneously HCV clearers HIV-elite controllers (nSC), 13 HCV spontaneous clearers non-HIV-elite controllers (SnC), and 15 non-spontaneously HCV clearers non-HIV-elite controllers (nSnC). HIV-elite controllers were defined as HIV-infected patients with VL < 50 HIV-RNA copies/mL in absence of antiretroviral treatment for at least 1 year. Non-HIV-elite controllers were patients with confirmed VL > 2 × 10^3^ HIV-RNA copies/mL in absence of antiretroviral treatment. HCV spontaneous clearers were defined as HCV infected patients (anti-HCV antibody positive) with < 10 HCV-RNA copies/ml in absence of anti-HCV treatment. Non-spontaneously HCV clearers or HCV chronic patients were those with confirmed detectable HCV VL, without anti-HCV treatment. This study was carried out in accordance with the recommendations of “Comité de Etica de la Investigación de Centro Hospital Universitario Virgen del Rocío de Sevilla” with written informed consent from all subjects. All subjects gave written informed consent in accordance with the Declaration of Helsinki. The protocol was approved by the Comité de Etica de la Investigación de Centro Hospital Universitario Virgen del Rocío de Sevilla (2012PI/240).

### Laboratory Measurements

Absolute counts of CD4+ and CD8+ T-cells were determined in fresh whole blood by using an Epic XL-MCL flow cytometer (Beckman-Coulter, Brea, CA) according to the manufacturer's instructions. The plasma HIV-1 RNA concentration was measured by using quantitative PCR (Cobas Ampliprep/Cobas TaqMan HIV-1 test; Roche Molecular Systems, Basel, Switzerland) according to the manufacturer's protocol. The detection limit for this assay was 50 HIV-1 RNA copies/mL. Hepatitis C virus (HCV) RNA was determined using an available PCR procedure kit (Cobas Amplicor; Roche Diagnostics, Barcelona, Spain) with a detection limit of 10 HCV-RNA copies/ml. Plasma samples were tested for anti-HCV antibodies using HCV-ELISA (Siemens Healthcare Diagnosis, Deerfield, IL, USA). HCV genotype was determined using a reverse-hybridization assay (InnoLIPA HCV II; Innogenetics, Barcelona, Spain).

### Cell Stimulation

Peripheral blood mononuclear cells (PBMCs) were thawed, washed and rested for 2h in DNase I (Roche Diagnostics, Indianapolis, IN)-containing R-10 complete medium. 1.5 × 10^6^ PBMCs including 10 μg/mL of brefeldin A (Sigma Chemical Co, St. Louis MO), and 0.7 μg/mL of monensin (BD Biosciences), were stimulated *in vitro* for 6 h with 2 μg/ml of each peptide pool of HCV NS4A, HCV NS4B, HCV NS3, and HCV Core (BEI Resources Repository, Manassas, VA, USA). HCV peptides were based on HCV 1a H77 sequence. In addition, 1.5 × 10^6^ PBMCs were stimulated with 2 μg/ml of an overlapped HIV (Gag)-specific peptide pool (NIH AIDS Reagent Program [https://www.aidsreagent.org/index.cfm]). 1.5 × 10^6^ unstimulated cells and cells stimulated with staphylococcal enterotoxin B (SEB) as a positive control were included in each experiment. The stimulation was performed in the presence of titrated amounts of anti-CD107a-BV605 (clone H4A3; BD Biosciences, USA) monoclonal antibody as previously described (18). T-cell specific response was defined as the frequency of cells with detectable intracellular cytokine production, after background subtraction of the unstimulated condition, after stimulation with HCV NS3, NS4A, NS4B, and Core peptide and HIV Gag peptides. For this analysis 1 × 10^6^ events were acquired and a median of 4.72 × 10^5^ live T-cells were gated.

### Immunophenotyping and Intracellular Cytokine Staining

Stimulated PBMCs were washed and stained with LIVE/DEAD fixable aqua dead cell stain (Life Technologies, CA, USA). The cells were then surface stained with anti-CD14-BB630, anti-CD20-BB630 (clones MoP9 and 2H7B, respectively, BD Bioscience, custom made), anti-CXCR3-BV421 (clone 1C6/CXCR3), anti-TIGIT-BV785 (clone 1G9), anti-CXCR6-BUV395 (clone 13B1E5), anti-CD56-BUV563 (clone NCAM16.2), anti-CD4-BUV805 (clone SK3) (BD Biosciences, USA), anti-Lag3-BV650 (clone 11C3C63), anti-PD1-BV711 (clone EH12.2H7), anti-CD161 (clone HP-3G10), anti-HLA-DR (clone L243) (Biolegend, USA), anti-Tim3-PE (clone FAB2356P, R&D), anti-CD45RO-ECD (clone UCHL1), anti-CD27-PECy5 (1A4CD27) (Beckman Coulter, USA) for 20 min at room temperature. Cells were then permeabilized (BD Cytofix/Cytoperm buffer, BD Bioscience, USA) and stained intracellularly with anti-CD3-BUV496 (clone UCHT7), anti-IFNγ-FITC (clone B27), anti-tumor necrosis factor alpha (TNFα)-PECy7 (clone MIH1), anti-IL2-APC (clone 5344.111) (BD Biosciences, USA), and anti-Granzyme B-PECy5.5 (clone GB11) (Thermo Fisher, USA) for 30 min at 4°C, and then washed twice and fixed in PBS containing 4% paraformaldehyde (PFA). Cells were acquired on a 30-parameters A5 Symphony flow cytometer using FACS Diva Software (BD Bioscience, Bethesda, USA). Data were analyzed using the FlowJo software (Treestar, Ashland, OR).

### Dendritic Cells Immunophenotyping

When samples were available, PBMCs were stained with zombie UV dye (Biolegend, USA) and surface stained with Lineage cocktail 3-FITC, anti-b7-BV605 (clone FIB504), anti-CD141-BV650 (clone 1A4), anti-CD103-BV711 (BerACT8), anti-CD83-BUV395 (clone HB15e), anti-CD16-BUV496 (clone 3G8), anti-CD56-BUV563 (NCAM16.2), anti-CD11c-BUV661 (clone B-ly6), anti-CD86-BUV737 (clone 2331), anti-CD4-BUV805 (clone SK3), anti-CCR7-Ax700 (clone 150502), anti-CCR5-APCCy7 (clone 2D7/CCR5), anti-CD40-PECy5 (clone 5C3), and anti-PDL1-PECy7 (clone MIH1) (BD Bioscience, USA), anti-CD123-BV421 (clone GH6), anti-CD1c-BV510 (clone LI61), anti-BDCA2-BV785 (clone 201A), anti-CCR2-APC (clone K036C2), and anti-CCR9-PE (clone L053E8) (Biolegend, USA), anti-CD2-PETexaRed (clone RPA-2.10), and anti-HLADR-PECy5.5 (clone TU36) (Thermo Fisher, USA) for 20 min at room temperature and then washed twice and fixed in PBS containing 4% PFA. Cells were acquired on a 30-parameters A5 Symphony flow cytometer using FACS Diva Software (BD Bioscience, Bethesda, USA); data were analyzed using the FlowJo software (Treestar, Ashland, OR).

### Statistical Analysis

Differences between unpaired samples were analyzed by Mann–Whitney *U*-tests. Correlations between variables were assessed using the Spearman rank test. All differences with a *P* < 0.05 were considered statistically significant. Statistical analyses were performed by using Statistical Package for the Social Sciences software (SPSS 22.0; SPSS Inc., Chicago, IL). Graphs were generated with Prism, version 5.0 (GraphPad Software, Inc.) and R Statistical Software (Foundation for Statistical Computing, Vienna, Austria) (19). Polyfunctionality was defined as the percentage of lymphocytes producing multiple cytokines. Polyfunctionality pie charts were constructed using Pestle version 1.6.2 and Spice version 5.2 (provided by M. Roederer, NIH, Bethesda, MD) and was quantified with the polyfunctionality index algorithm (20) employing the 0.1.2 beta version of the FunkyCells Boolean Dataminer software provided by Martin Larson (INSERM U1135, Paris, France). Differences between unpaired distributions in pie charts were analyzed by Permutation test, Spice version 5.2. In this test a *P* < 0.05 was considered statistically significant.

## Results

### Patients' Characteristics

Patients' characteristics are summarized in Table 1. No differences were found in sex, time from HIV diagnosis and category of transmission among groups. HIV-controllers were slightly older than non-controllers (53 [52–54] years old of SC and 47 [42–53] years old of nSC vs. 41 [36–46] years old for SnC and 45 [40–49] years old for nSnC). As expected, HIV controller groups, SC (supercontrollers) and nSC (non-spontaneously HCV clearers HIV-elite controllers) had higher nadir and CD4 T-cells levels (438 [281–598] cells/μl of SC and 550 [329–698] cells/μl of nSC) than non-controller groups, SnC (HCV spontaneous clearers non-HIV-elite controllers, 114 [35–295] cells/μl) and nSnC (non-spontaneously HCV clearers non-HIV-elite controllers, 39 [11–169] cells/μl).

**Table 1:** Patient's characteristics.

**ID**	**Sex**	**Age (years)**	**Time from HIV diagnosis (years)**	**Risk trasmission**	**nadir**	**CD4 T cells (cells/μl)**	**CD8 T cells (cells/μl)**	**HIV VL (log copies/ml)**	**HCV VL (log copies/ml)**	**HCV genotype**
SC 1	Male	53	22.66	IDU	586	987	829	ND	ND	
SC 2	Male	52	17.40	IDU	114	114	210	ND	ND	
SC 3	Male	52	26.60	IDU	248	248	258	ND	ND	
SC 4	Male	59	25.86	IDU	380	380	880	ND	ND	
SC 5	Male	41	20.53	IDU	418	640		ND	ND	
SC 6	Female	54	18.49	HTX	459	459	398	ND	ND	
SC 7	Female	53	19.45	IDU	603	850		ND	ND	
SC 8	Female	53	28.08	IDU	677	1076	816	ND	ND	
nSC 1	Female	43	18.86	HTX	226	447	902	ND	7.03	1
nSC 2	Female	38	19.05	IDU	669	1155	484	ND	4.53	4
nSC 3	Female	59	28.68	HTX	238	350	193	ND	6.70	4
nSC 4	Male	46	0.75	IDU	703	901	822	ND	6.19	
nSC 5	Male	32	1.97	IDU	697	759		ND	5.85	
nSC 6	Male	53	11.97	IDU	360	660	960	ND	5.69	1
nSC 7	Female	41	22.68	IDU	211	682	263	ND	4.82	4
nSC 8	Male	48	22.45	IDU	559	635	880	ND	5.88	1
nSC 9	Male	54	0.16	IDU	459	1010	332	ND	6.60	3
nSC 10	Male	45	21.33	IDU	541	978	1013	ND	6.74	1
nSC 11	Female	54	26.08	IDU	407	1028	415	ND	5.25	
nSC 12	Female	45	0.07	HTX	1071	1071	589	ND	6.01	
nSC 13	Male	49	20.34	IDU	840	1127		ND	6.43	3
nSC 14	Male	52	30.37	IDU	675	1067	830	ND	6.05	1
SnC 1	Male	38	11.44	IDU	103	103	668	5.12	ND	
SnC 2	Male	41	14.35	IDU	279	368	875	4.67	ND	
SnC 3	Female	38	15.33	IDU	311	313	976	4.39	ND	
SnC 4	Male	33	7.44	IDU	6	23	674	5.01	ND	
SnC 5	Male	42	1.91	IDU	39	39	827	5.68	ND	
SnC 6	Male	41	17.92	IDU	261	261	774	4.77	ND	
SnC 7	Female	45	12.10	HTX	390	592	600	4.05	ND	
SnC 8	Male	34	9.16	IDU	253	362	1032	3.93	ND	
SnC 9	Male	38	16.80	HTX	46	46	819	5.33	ND	
SnC 10	Male	50	23.42	IDU	12	26	564	4.81	ND	
SnC 11	Male	30	0.52	IDU	323	408	1293	3.35	ND	
SnC 12	Male	51	24.44	IDU	114	133	437	4.23	ND	
SnC 13	Male	48	25.35	IDU	31	196	1390	5.38	ND	
nSnC 1	Female	37	14.09	IDU	106	172	579	6.14	6.00	1
nSnC 2	Male	40	23.69	IDU	33	33	106	5.81	7.20	1
nSnC 3	Male	40	15.76	IDU	27	149	616	5.27	5.45	1
nSnC 4	Female	39	15.27	HTX	169	169	438	4.64	5.97	1
nSnC 5	Male	41	20.59	IDU	7	7	136	5.36	8.00	1
nSnC 6	Male	45	18.88	IDU	237	290	758	4.97	6.70	1
nSnC 7	Male	47	17.82	MSM	230	256	775	5.13	6.16	1
nSnC 8	Male	49	18.80	IDU	21	45	512	5.41	7.51	3
nSnC 9	Male	42	24.11	IDU	39	39	2436	4.29	7.52	1
nSnC 10	Male	49	23.92	IDU	157	157	2098	5.42	7.88	1
nSnC 11	Female	44	20.42	IDU	3	13	264	5.23	7.22	1
nSnC 12	Male	46	22.35	IDU	7	34	696	5.36	5.25	1
nSnC 13	Female	50	25.46	IDU	456	799	950	4.49	6.76	3
nSnC 14	Male	49	17.37	IDU	11	389	761	4.66	6.46	1
nSnC 15	Male	53	19.99	IDU	112	188	1348	5.74	5.79	1

*ND, not detectable; IDU, intravenous drug user; HTX, heterosexual contact; MSM, men who have sex with men. HCV genotype was not detectable in HCV spontaneous clearers and in 3 of the nSC. SC, supercontrollers; HCV spontaneous clearers and HIV-elite controllers; nSC, non-spontaneously HCV clearers HIV-elite controllers; SnC, HCV spontaneous clearers non-HIV-elite controllers; nSnC, non-spontaneously HCV clearers non-HIV-elite controllers*.

### Improved Polyfunctionality of HCV- and HIV-Specific CD4 T-Cells in Supercontrollers

**Figure 1:**
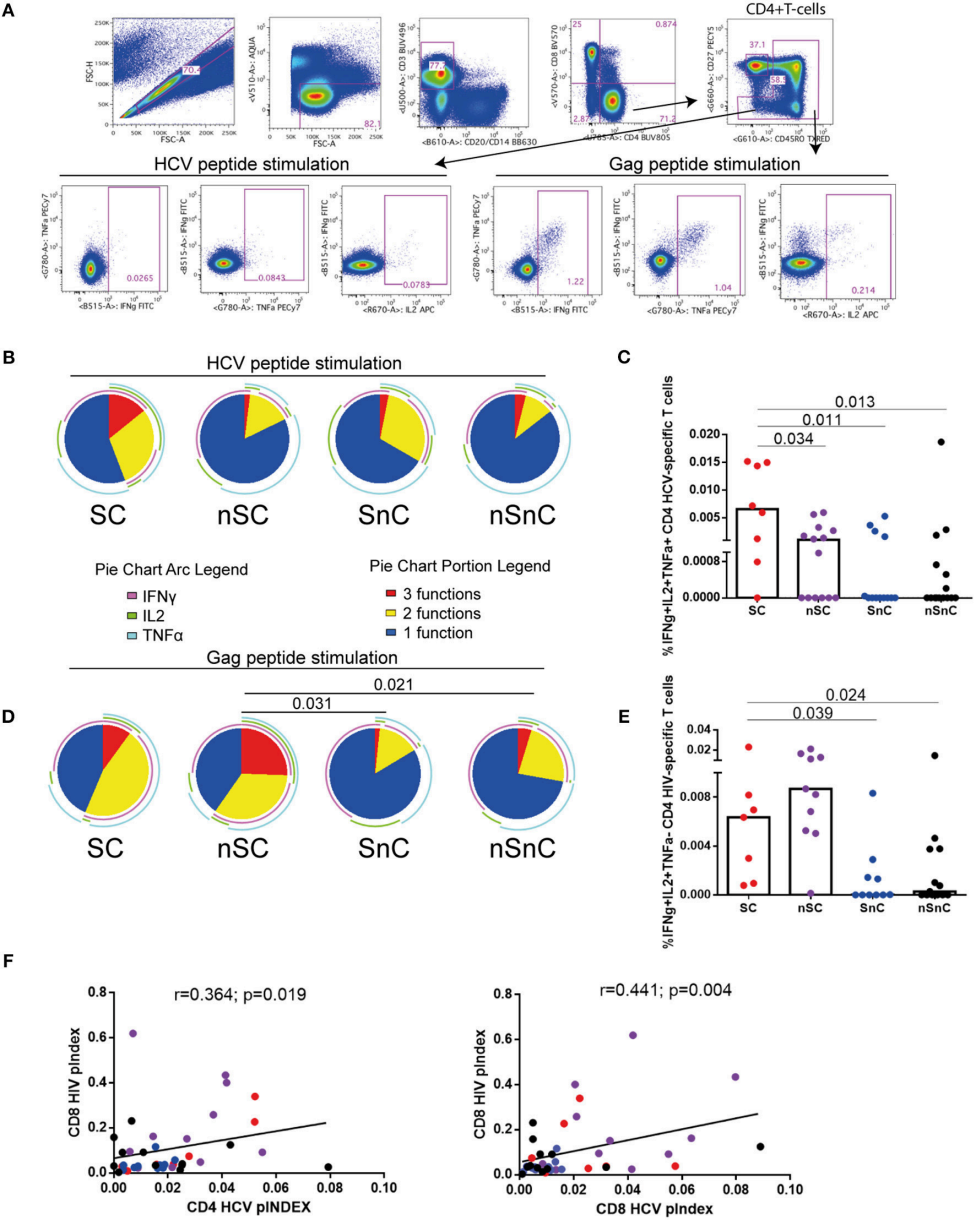
HCV- and HIV- CD4 specific T-cells. **(A)** gating stratregy. **(B)** HCV-specific CD4 T-cell polyfunctionality pie charts. Permutation test, following the Spice version 5.2 software was used to assess diferencies between pie charts. **(C)** Cytokines combinations from HCV-specific CD4 T-cell. Mann–Whitney *U*-tests was used to assess diferencies between groups. **(D)** HIV-specific CD4 T-cell polyfunctionality pie charts. Permutation test, following the Spice version 5.2 software was used to assess diferencies between pie charts. **(E)** Cytokines combinations from HIV-specific CD4 T-cell. Mann–Whitney *U*-tests was used to assess diferencies between groups. **(F)** Spearman correlations between CD4 and CD8 HCV-specific T-cells polyfunctionality index and CD8 HIV-specific T-cells polyfunctionality index. SC: supercontrollers, HCV spontaneous clearers and HIV-elite controllers; nSC: non-spontaneously HCV clearers HIV-elite controllers; SnC: HCV spontaneous clearers non-HIV-elite controllers; nSnC: non-spontaneously HCV clearers non-HIV-elite controllers.

We analyzed the T-cell specific response defined as the frequency of cells (after background subtraction of the unstimulated condition) with detectable intracellular cytokine production after stimulation with HCV NS3, NS4A, NS4B, and Core peptide and HIV Gag peptides. CD4 HCV and HIV specific T-cell gating strategies are shown in Figure 1A. T-cells from SC exhibited the highest HCV-specific CD4 T-cell polyfunctionality, in terms of simultaneously production of IFNγ, IL2, and TNFα (Figure 1B, red portion of pie chart). Although the overall distribution of cytokine production was not statistically significant, when we analyzed the proportion of CD4 T-cells expressing IFNγ, IL2, and TNFα (3 functions) at the same time in response to HCV, the highest levels were shown in SC group (Figure 1C). Interestingly, this cell subset was higher in SC than SnC, the group of patients that spontaneously clear the HCV. When the HIV-specific CD4 T-cell response was analyzed, a higher polyfunctionality was present in HIV-controllers, independently of HCV clearance (SC and nSC groups) (Figure 1D, red portions). Also, the frequency of HIV specific CD4 T-cells producing the combination IFNγ+IL2+TNFα- was higher in the HIV-controller groups (SC and nSC) respect non-HIV- controller groups (SnC and nSnC) (Figure 1E). The CD4 and CD8 T-cell polyfunctionality index (pINDEX) in response to HCV positively correlated with the CD8 T-cell pINDEX in response to HIV (*r* = 0.364, *p* = 0.019; and *r* = 0.441, *p* = 0.004, respectively) (Figure 1F), reinforcing the idea of the better T-cell response to HCV corresponded with a better T-cell response to HIV. When CD4 T-cell cytotoxicity was analyzed, assessed by CD107a and Granzyme B production in any combination with other cytokine, no differences were observed in response to HCV and HIV stimulation (data not shown). Interestingly, controllers (SC and nSC) exhibited higher CD107a+GranzymeB-IFNg-IL2-TNFa- CD4 HIV-specific T-cells than non-controllers (Supplementary Figure 1). HCV- and HIV-specific CD8 T-cell response was also analyzed (Supplementary Figure 2). Gating strategies are summarized in Supplementary Figure 2A. No significant differences in HCV- and HIV-specific CD8 T-cell response among groups were observed (Supplementary Figure 2B). We observed differences in some cytokine combination, such as higher proportion of IFNγ+IL2-TNFα+ CD8 HCV-specific T-cells of SC compared with SnC and nSnC (*p* = 0.025 and 0.049, respectively) (Supplementary Figure 2C). Interestingly, we observed a trend to higher CD8 HIV-specific T-cells polyfunctionality (Supplementary Figure 2D) and higher proportion of IFNγ+IL2-TNFα+ and IFNγ+IL2+TNFα+ CD8 HIV-specific T-cells (Supplementary Figure 2E) in nSC group than SnC and nSnC.

### Low Levels of T-Cell Exhaustion in HIV-Controllers Independently of HCV Clearance

**Figure 2:**
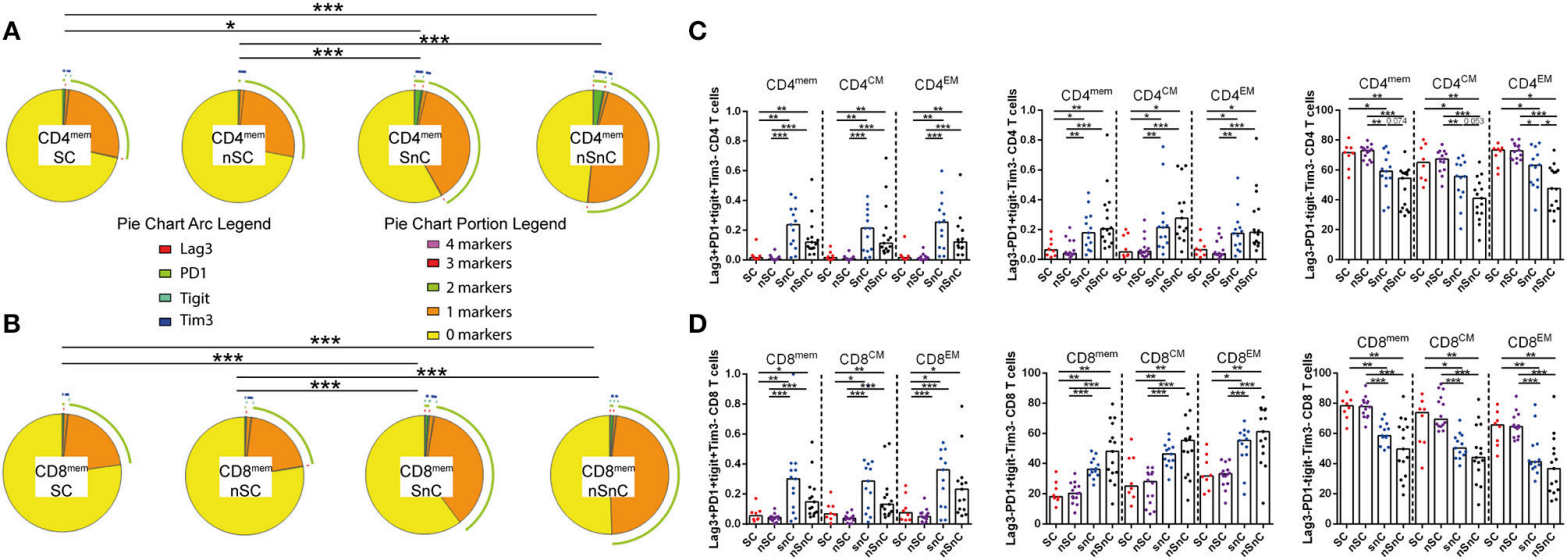
T-cell exhaustion. **(A)** multiple exhaustion phenotype from CD4 memory T-cells pie charts. Permutation test, following the Spice version 5.2 software was used to assess diferencies between pie charts. **(B)** multiple exhaustion phenotype from CD8 memory T-cells pie charts. Permutation test, following the Spice version 5.2 software was used to assess diferencies between pie charts. **(C)** Combinations of exhaustion markers within CD4 memory subsets. Mann–Whitney *U*-tests was used to assess diferencies between groups. **(D)** Combinations of exhaustion markers within CD8 memory subsets. Mann–Whitney *U*-tests was used to assess diferencies between groups. mem, total memory; CM, central memory; EM, effector memory. SC, supercontrollers, HCV spontaneous clearers and HIV-elite controllers; nSC, non-spontaneously HCV clearers HIV-elite controllers; SnC, HCV spontaneous clearers non-HIV-elite controllers; nSnC, non-spontaneously HCV clearers non-HIV-elite controllers. Statistical values are shown as: ^*^p = 0.05–0.01; ^**^p = 0.01–0.001; ^***^p < 0.001.

To determine whether HCV-specific CD4 T-cell polyfunctionality was associated to lower T-cell exhaustion we quantified the expression of the exhaustion markers Lag3, PD1, TIGIT, and Tim3 in CD4 and CD8 memory T-cell subsets (gating strategy is shown in Supplementary Figure 3). The “multiple exhausted phenotype” (simultaneous expressions of three or more of the analyzed exhaustion markers) was represented by pie charts. SC exhibited low multiple exhausted phenotypes in both CD4 and CD8 T-cells, in all memory subpopulations. These levels were similar to the other HIV-controller group, nSC (Figures 2A,B). This trend was observed when specific combinations of exhaustion markers were analyzed in each group (Figure 2C). Regarding CD8 T-cell subsets similar patterns to CD4 T-cell subsets were shown when exhaustion marker combinations were analyzed (Figure 2D).

### T-Cell Activation and CD161^high^ CD8 T-Cell Correlated With HCV and HIV T-Cell Response

**Figure 3:**
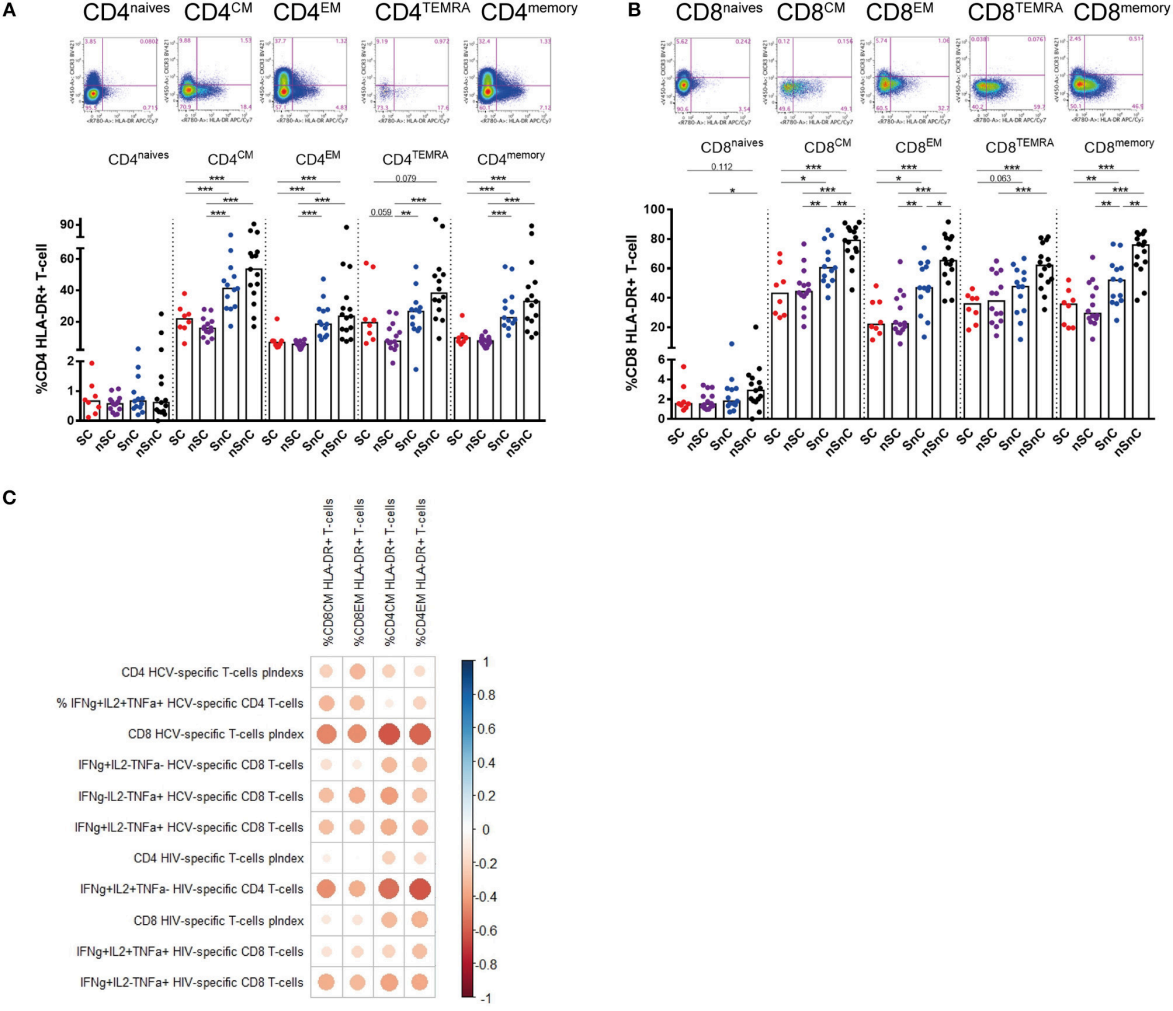
T-cell activation. **(A)** Frequency of CD4 T-cell subsets expressing HLA-DR. Mann–Whitney *U*-tests was used to assess diferencies between groups. **(B)** Frequency of CD8 T-cell subsets expressing HLA-DR. Mann–Whitney *U*-tests was used to assess diferencies between groups. **(C)** Pearson correlation between CD4 and CD8 expressing HLA-DR and HCV- and HIV-specific T-cell responses. mem, memory total; CM, central memory; EM, effector memory; TEMRA, terminally differenciated. SC, supercontrollers, HCV spontaneous clearers and HIV-elite controllers; nSC, non-spontaneously HCV clearers HIV-elite controllers; SnC, HCV spontaneous clearers non-HIV-elite controllers; nSnC, non-spontaneously HCV clearers non-HIV-elite controllers. Statistical values are shown as: ^*^p = 0.05–0.01; ^**^p = 0.01–0.001; ^***^p < 0.001. The ball size corresponds with the magnitude of Pearson's *r*-value.

**Figure 4:**
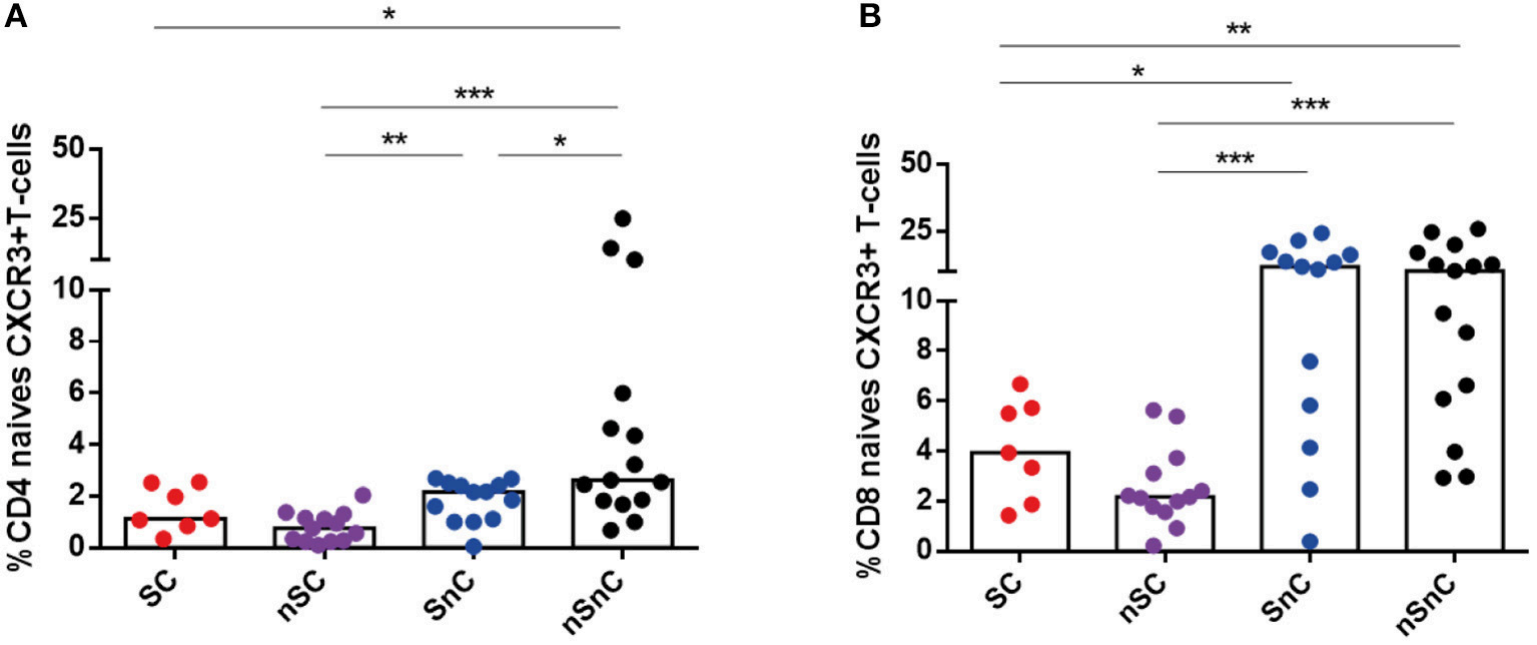
CXCR3 expression in naive CD4 and CD8 T-cells. **(A)** Frequency of naive CD4 CXCR3+ T-cells. **(B)** Frequency of naive CD8 CXCR3+ T-cells. One outlyer SC and one nSC were excluded. SC, supercontrollers, HCV spontaneous clearers and HIV-elite controllers; nSC, non-spontaneously HCV clearers HIV-elite controllers; SnC, HCV spontaneous clearers non-HIV-elite controllers; nSnC, non-spontaneously HCV clearers non-HIV-elite controllers. Mann–Whitney *U*-tests was used to assess diferencies between groups. Statistical values are shown as: ^*^p = 0.05–0.01; ^**^p = 0.01–0.001; ^***^p < 0.001.

T-cell activation was assessed by HLA-DR expression both in CD4 and CD8 T-cell subpopulations. SC presented lower CD4 HLA-DR+ T-cell levels (Figure 3A) and CD8 HLA-DR T-cells (Figure 3B) in every subpopulation studied and, similarly to T-cell exhaustion, at the same levels than the other HIV controller group (nSC). Interestingly, this activated phenotype of CD8 and CD4 central and effector memory T-cells showed a common pattern of negative correlations with CD4 and CD8 HCV-and HIV-specific T-cell responses (Figure 3C). In addition, the chemokine receptor CXCR3, involved in T-cell trafficking to inflamed tissue (21) was also analyzed in CD4 and CD8 T-cells. We did not found differences in CXCR3 expression in any memory subset, neither in CD4 nor CD8 T-cell (data not shown). Unexpectedly, we observed lower levels of CXCR3+ naïve CD4 and CD8 T-cells in HIV-controllers (SC and nSC) compared with non-HIV controllers (SnC and nSnC) (Figures 4A,B).

**Figure 5:**
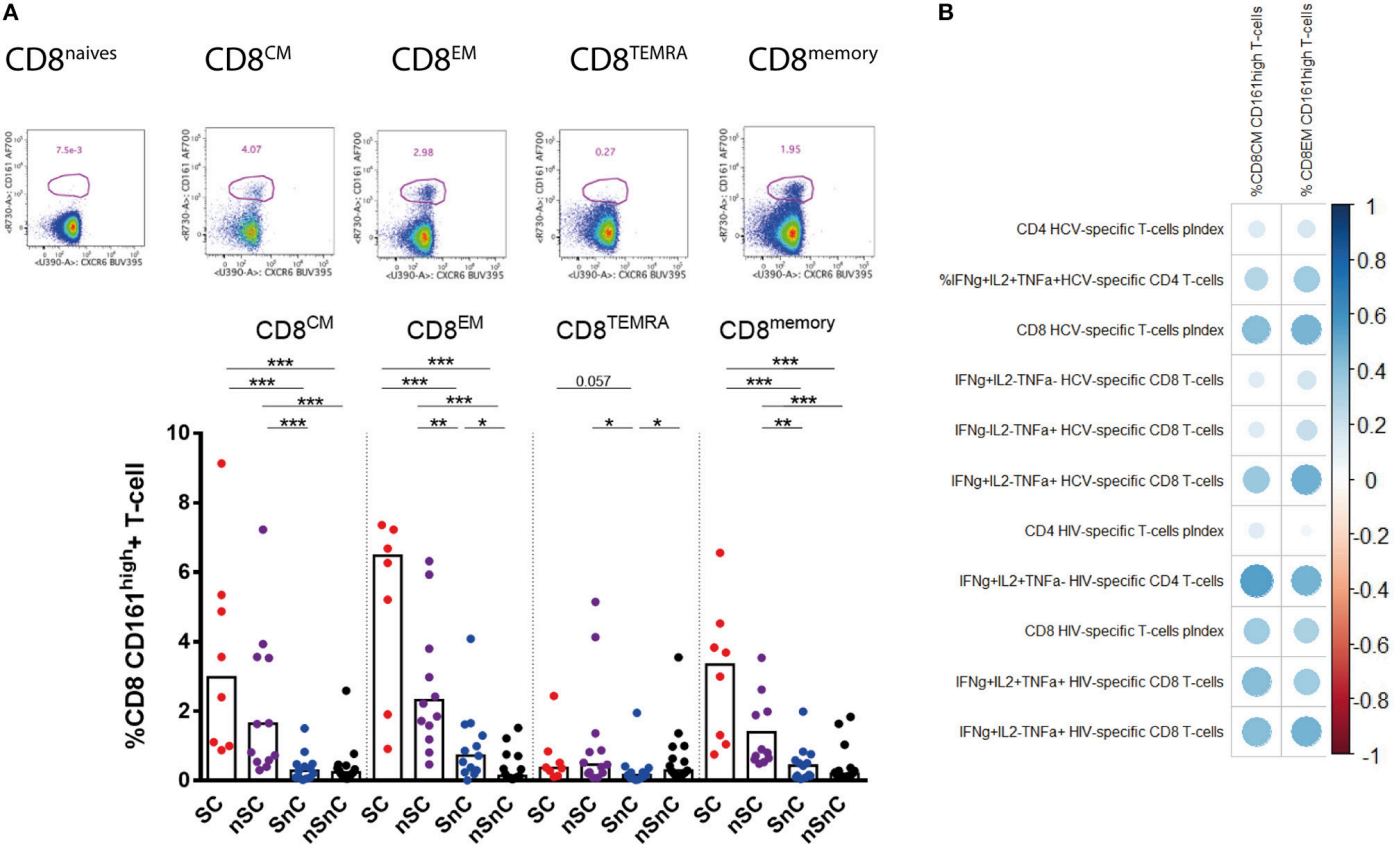
CD161^high^ CD8 T-cells. **(A)** Frecuency of CD161^high^ among CD8 T-cells subsets. Mann–Whitney *U*-tests was used to assess diferencies between groups. **(B)** Pearson correlation between CD161^high^ CD8 T-cells and HCV- and HIV-specific T-cell responses. SC, supercontrollers, HCV spontaneous clearers and HIV-elite controllers; nSC, non-spontaneously HCV clearers HIV-elite controllers; SnC, HCV spontaneous clearers non-HIV-elite controllers; nSnC, non-spontaneously HCV clearers non-HIV-elite controllers. Statistical values are shown as: ^*^p = 0.05–0.01; ^**^p = 0.01–0.001; ^***^p < 0.001. The ball size correspond with the magnitude of Pearson's *r*-value.

The expression of the C-type lectin CD161 was also analyzed in CD4 and CD8 T-cells. No significant differences in CD161 expression of CD4 T-cells were found among groups (data not shown). Higher frequency of CD161^high^ CD8 T-cell was presented in HIV-controllers, SC and nSC, compared with non-HIV controllers, SnC and nSnC (Figure 5A). Notably, we observed higher frequency of CD161^high^ CD8 effector memory T-cells in SnC than nSnC. This peculiar CD8 T-cell phenotype from central and effector memory subsets strongly correlated with HCV-and HIV-specific CD4 and CD8 T-cell responses (Figure 5B).

### Innate Immune Cells Involvement in HIV Control and HCV Spontaneous Clearance

**Figure 6:**
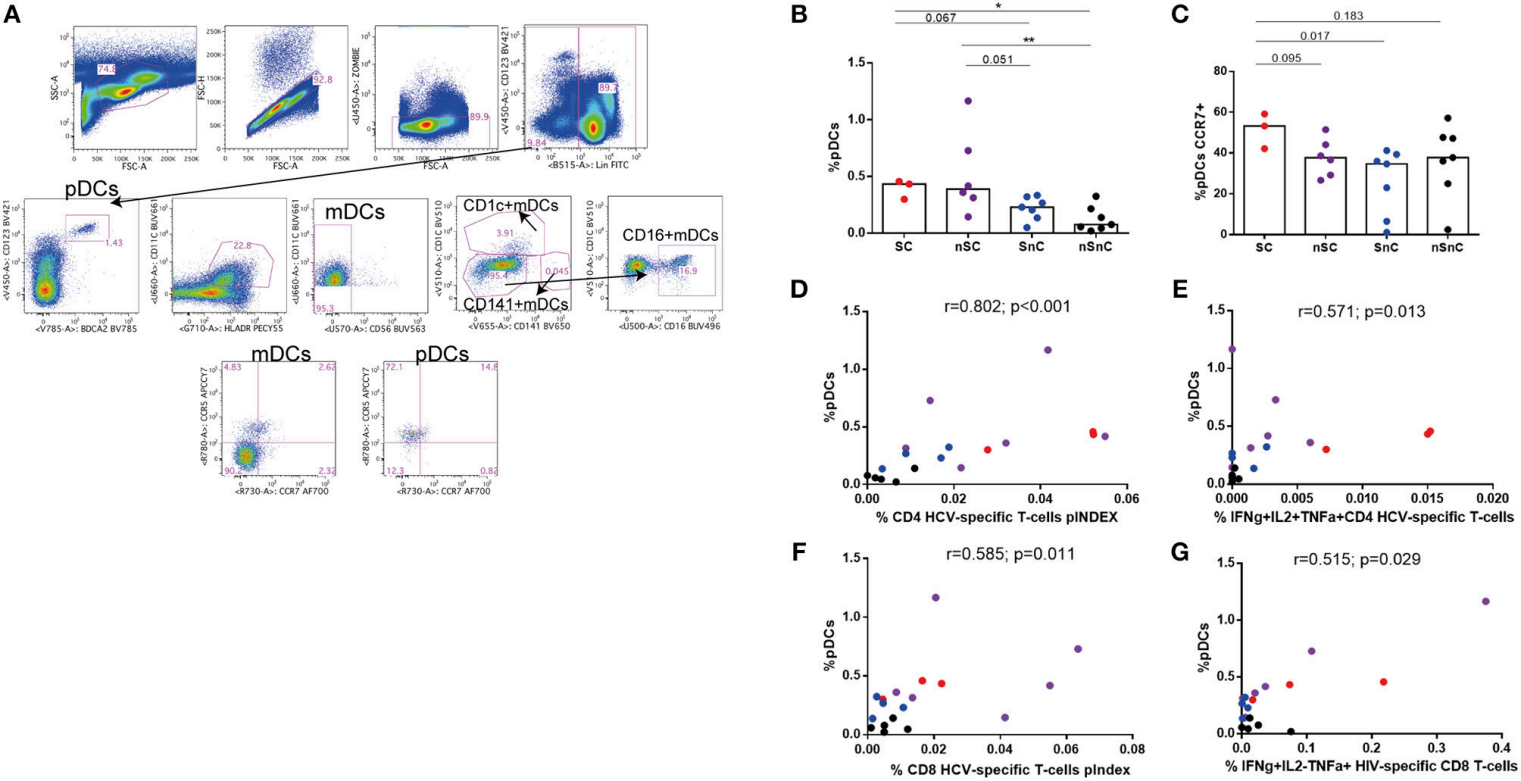
Dendritic cells. **(A)** Gating strategy. **(B)** Plasmacytoid dendritic cells (pDCs) frequency. Mann–Whitney *U*-tests was used to assess diferencies between groups. **(C)** CCR7 expresing pDCs. Mann–Whitney *U*-tests was used to assess diferencies between groups. **(D-G)** Spearman correlations between pDCs frequency and HCV- and HIV-specific T-cell responses. SC, supercontrollers, HCV spontaneous clearers and HIV-elite controllers; nSC, non-spontaneously HCV clearers HIV-elite controllers; SnC, HCV spontaneous clearers non-HIV-elite controllers; nSnC, non-spontaneously HCV clearers non-HIV-elite controllers. Statistical values are shown as: ^*^p = 0.05–0.01; ^**^p = 0.01–0.001; ^***^p < 0.001.

We then ought to analyze the association of innate immunity in the supercontroller phenotype. The gating strategies for different dendritic cell subsets are summarized in Figure 6A. As expected, higher frequency of pDCs were found in HIV-controllers (SC and nSC) compared with non-controllers (SnC and nSnC), independently of HCV spontaneous clearance (Figure 6B). No differences in mDCs were found among groups (data not shown). Interestingly, there was a trend to show higher frequency of pDCs expressing the lymph node homing marker CCR7 in SC (Figure 6C). Furthermore, positive correlations were found between frequency of pDCs and HCV-specific CD4 T-cell pINDEX (*r* = 0.802, *p* < 0.001) (Figure 6D) and frequency of IFNγ+IL2+TNFα+ CD4 HCV-specific T-cells (the combination shown in Figure 1C) (*r* = 0.571, *p* = 0.013) (Figure 6E). Also, positive correlations were found between pDCs levels and HCV-specific CD8 T-cell pINDEX (*r* = 0.585, *p* = 0.011) (Figure 6F) and frequency of IFNγ+IL2-TNFα+ HIV-specific CD8 T-cells (*r* = 0.515, *p* = 0.029) (Figure 6G).

**Figure 7:**
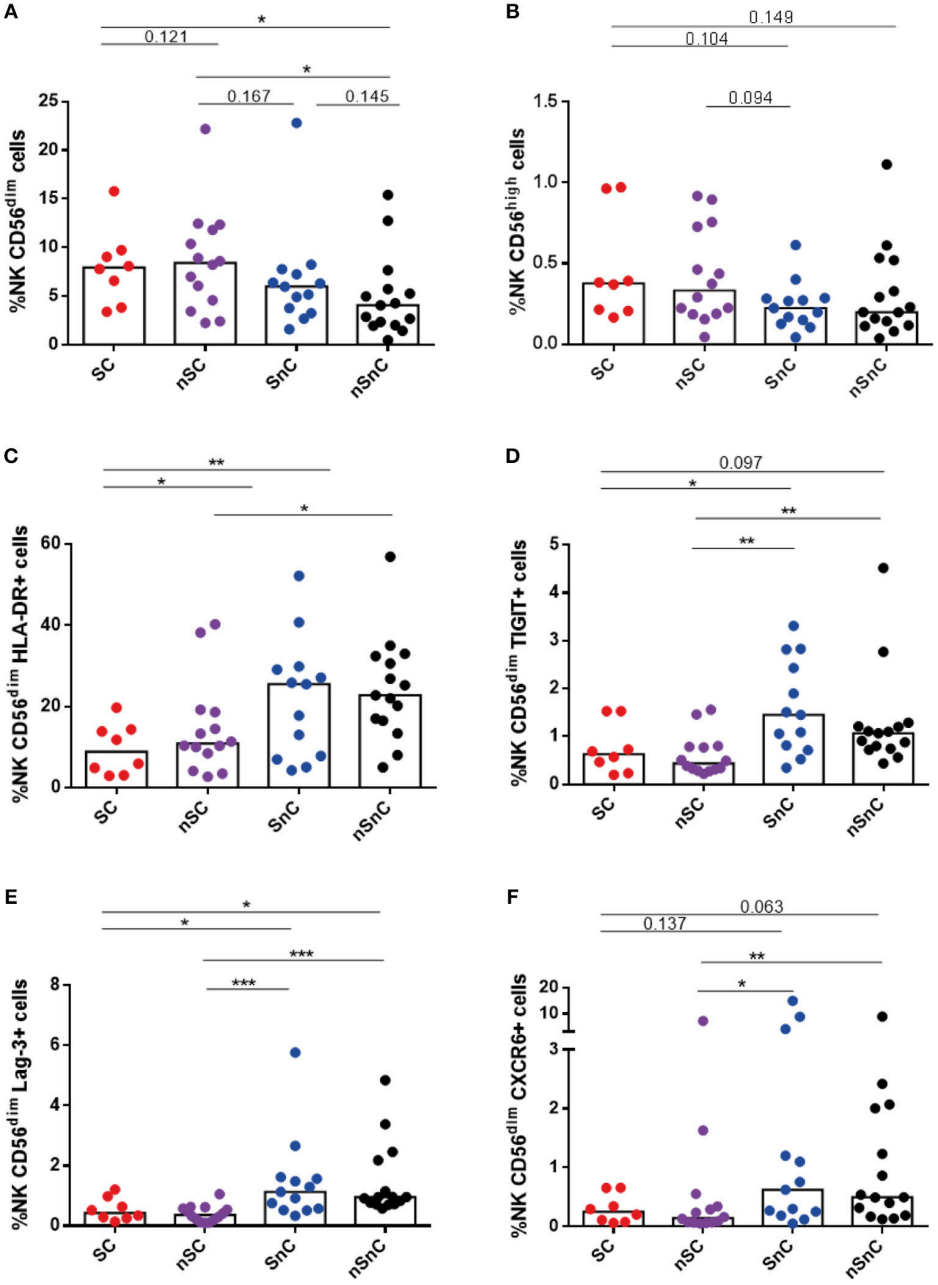
NK cels. **(A)** NK CD56^dim^ cells frequency and **(B)** NK CD56^high^ cells frequency. **(C–E)** NK CD56^dim^ cells expressing HLA-DR, Lag-3 and TIGIT. **(F)** NK CD56^dim^ cells expressing CXCR6. SC, supercontrollers, HCV spontaneous clearers and HIV-elite controllers; nSC, non-spontaneously HCV clearers HIV-elite controllers; SnC, HCV spontaneous clearers non-HIV-elite controllers; nSnC, non-spontaneously HCV clearers non-HIV-elite controllers. Mann–Whitney *U*-tests was used to assess diferencies between groups. Statistical values are shown as: ^*^p = 0.05–0.01; ^**^p = 0.01–0.001; ^***^p < 0.001.

Regarding NK cells, we also observed higher levels of NK CD56^dim^ cells among HIV-controller group, SC and nSC (Figure 7A). No differences of NK CD56^high^ cells were found among groups (Figure 7B). In line with the results for CD4 and CD8 T-cell activation, HIV controller groups, SC and nSC, presented lower levels of NK CD56^dim^ cells expressing HLA-DR (Figure 7C), likewise some exhaustion markers such as TIGIT (Figure 7D) and Lag3 (Figure 7E), and CXCR6 (Figure 7F). Gating strategies of NK cells are shown in Supplementary Figure 4.

## Discussion

HIV-elite controllers that spontaneously clear HCV (supercontrollers) are extremely difficult to investigate, as they are very uncommon. Considering that about 1% of patients are able to control HIV (22), and 10–20% of infected patients spontaneously clear the HCV (23), the proportion of patients that control both viruses is very low (we estimates < 0.2%). Herein, we have described for the first time the immunological features of these abovementioned supercontrollers, consisting in high CD4 HCV-specific T-cell polyfunctionality together with low exhausted and activated phenotype of T- and innate immunity cells.

Virus specific CD4 T-cells have an important role in controlling viral infections, not only by helping the CD8 T-cell cytotoxic activity and protective antibodies production by B cells, but also exercising antiviral functions (24, 25). We have found higher HCV-specific CD4 T-cell polyfunctionality in subjects that control HIV and clear HCV at the same time. That was in line with the only study of HCV T-cell response within HIV-controllers, in which the authors found a strong Th1 response against HCV (26). This study was performed with chronic HCV patients, not with HCV clearers, but an inverse correlation between Th1 frequency specific for HCV core protein and HCV VL was found (26). That emphasized the importance of CD4 T-cells in HCV control within HIV-controllers.

Interestingly, a higher, broadly and long lasting HCV-specific CD4 T-cell response has been associated with spontaneous clearance of HCV compared with chronic HCV patients (27–32). Also remarkably, these studies found broad CD4 T-cell responses to HCV non-structural protein NS3 and NS4 in HCV clearers, peptides that we have included in our study in the peptide pool for stimulations. Furthermore, Shulze et al. demonstrated that peptide recognized by CD4 T-cells from HCV spontaneous clearers were highly promiscuous, which can be restricted by multiple MHC-II molecules (32). Curiously, same phenomenon seems to occur with Gag peptide restriction in HIV-controllers, which same peptide can be restricted by up to five HLA-DR allomorphs to one T-cell receptor (33). Actually, several studies have associated some HLA-DRB1 alleles (34–36) and HLA-DQB1 (34, 37, 38) with HCV spontaneous clearance. Unfortunately, because of samples unavailability after flow cytometry experiments we were unable to test HLA-II typing. However, due to the small sample size this important immune correlate of virus control may be confirmed in larger cohorts of supercontrollers.

On the other hand, CD4 T-cell response were also related with HIV spontaneous control (39). It is known that HIV-controllers exhibit more vigorous, proliferative and polyfunctional CD4 HIV-specific T-cell responses than non-controller patients, even after the antiretroviral treatment onset (40–44). Furthermore, Potter et al. showed that CD4 T-cells from HIV-elite controllers were enriched of central memory, which expressed higher levels of CD127. This fact could contribute to a long-term antiviral memory activity in these patients (44), probably including HIV- and HCV-specific response. In our experiments, the CD4 HIV-specific response was similar in both HIV controller groups.

We did not observe significant differences in polyfunctionality of HCV-specific CD8 T-cell response among groups. It has been demonstrated that HCV-specific CD8 T-cell in chronic patients is difficult to find in peripheral blood, due to liver accumulation of these cells (45). Of note, although both SC and SnC spontaneously cleared the HCV, a higher IFNγ and TNFα HCV-specific CD8 T-cell production were found in patients that control HIV and clear HCV. However, we highlight the importance of the CD4 HCV-specific polyfunctionality found in SC. First, because a HCV-specific CD8 T-cell activity is known not to be enough to mount a protective response, as it has been demonstrate in the setting of HCV vaccines (46), probably because of the high scape mutation rate in this virus (47, 48). And second, because the importance of developing adequate CD4 T-cell response over a CD8 T-cell has been demonstrated in an elegant experiment with the HCV chimpanzee model in which Grakoui et al. depleted CD4 T-cells from animals that previously cleared the HCV, and then they reinfected them with HCV thus, demonstrating that despite of having memory HCV-specific CD8 T-cells, it was not enough to control the virus scape mutations onset (47).

T-cell exhaustion and activation are hallmarks of the HIV infection (49, 50). Interestingly, SC exhibited low levels of both measurements. Interestingly, CD4+ T-cell expressing PD1, Lag-3 and TIGIT has been strongly correlated with HIV reservoir size and HIV persistence (50). Maybe, this extraordinary group of controllers is enriched in patients with in addition to HCV spontaneous eradication, have minimal amount of integrated HIV. Unfortunately, we could not measure HIV reservoir because of lack of samples. Further analyses in this regard should be done. In this sense, Banga et al. argue that CD4 T-cell expressing CXCR3 represent the major blood compartment with HIV proviral population (51). No differences in CXCR3 expression in CD4 and CD8 T-cell memory subsets were found, but we observed lower frequency of naïve CD4 and CD8 T-cell expressing CXCR3 in HIV-controllers. This molecule is involved in T-cell homing to inflamed peripheral tissue after maturation (21). We did not expect to find CXCR3 expression in naive T-cells but although the expression were low (< 5% in HIV-controller groups), we observed differences with the non-controllers groups. We do not have a plausible explanation for this fact; maybe this is putative marker of lower inflammation/activation levels in HIV-controllers.

Interestingly, we found higher frequency of CD161^high^ CD8 T-cells in HIV-controllers, with a trend to high levels in SC. The significance of CD161 expression in CD8 T-cell is controversial (52). It has been suggested that CD161^high^ CD8 T-cell population represents a memory stem cell subset (53), whereas other authors argue that these cells are IL-17 producing CD8 T-cells (54, 55). Our results suggest that this CD8 T-cell subset could be involved somehow with the antiviral response (56), as they positively correlate with HIV- and HCV-specific T-cell responses.

Innate immunity also has a major role on viral control in both HIV and HCV (57, 58). We have previously communicated that pDCs are related with HIV spontaneous control (13, 14), and other authors showed higher responsiveness of pDCs from HCV spontaneous clearers (16). In this work we found higher pDCs frequency in HIV-controllers compared with non-controllers and interestingly, a trend to higher CCR7 expression of pDCs in SC. CCR7 is a homing molecule that enable pDC migrate to lymph nodes (59) and prime CD4 and CD8 T-cells (60). This is in agreement with the correlation found between pDCs and HIV- and HCV-specific T-cell levels. On the other hand, NK cells also play an important role in HIV control (9, 10) and HCV clearance (11, 12). Similarly to T-cells, HIV-controllers showed lower activation and exhaustion of CD56^dim^ NK cells compare with non-controllers, the high cytotoxic NK subset (61).

In conclusion, CD4 T-cell response seems to be the most differential feature in SC. Furthermore, this extraordinary group of subjects exhibited low levels of T-cell exhaustion and activation. Other T-cell subsets, as CD161^high^ were interestingly related with HIV and HCV-specific T-cell responses, as well as pDCs. Further analyses with a large cohort of SC must be done to deeply analyze HIV- and HCV-specific T-cell phenotype and the correlates with the control of both viruses in relation with other players of adaptive and innate immunity. The study of these patients could serve as a model for the development of therapeutic strategies aimed to enhance antiviral responses.

## Author Contributions

BD-M designed and performed experiments, analyzed and interpreted the data, designed the figures, and wrote the manuscript. SF-M and LT-D participated in the methodology and data analysis. JH-Q, MG, FV, and ML collaborated with the patient's characterization and samples collection. ML, MM-F, and RK supervised all the experimental procedures. ER-M and ML conceived and designed the study. ER-M interpreted the data and wrote the manuscript. All the authors critically reviewed, edited and approved the final manuscript.

### Conflict of Interest Statement

The authors declare that the research was conducted in the absence of any commercial or financial relationships that could be construed as a potential conflict of interest.
