# Supplementary figures and images for: Immune Correlates of Natural HIV Elite Control and Simultaneous HCV Clearance—Supercontrollers
Source: Front Immunol. 2018 Dec 10;9:2897. doi: 10.3389/fimmu.2018.02897 (PMC6295470; doi:10.3389/fimmu.2018.02897)

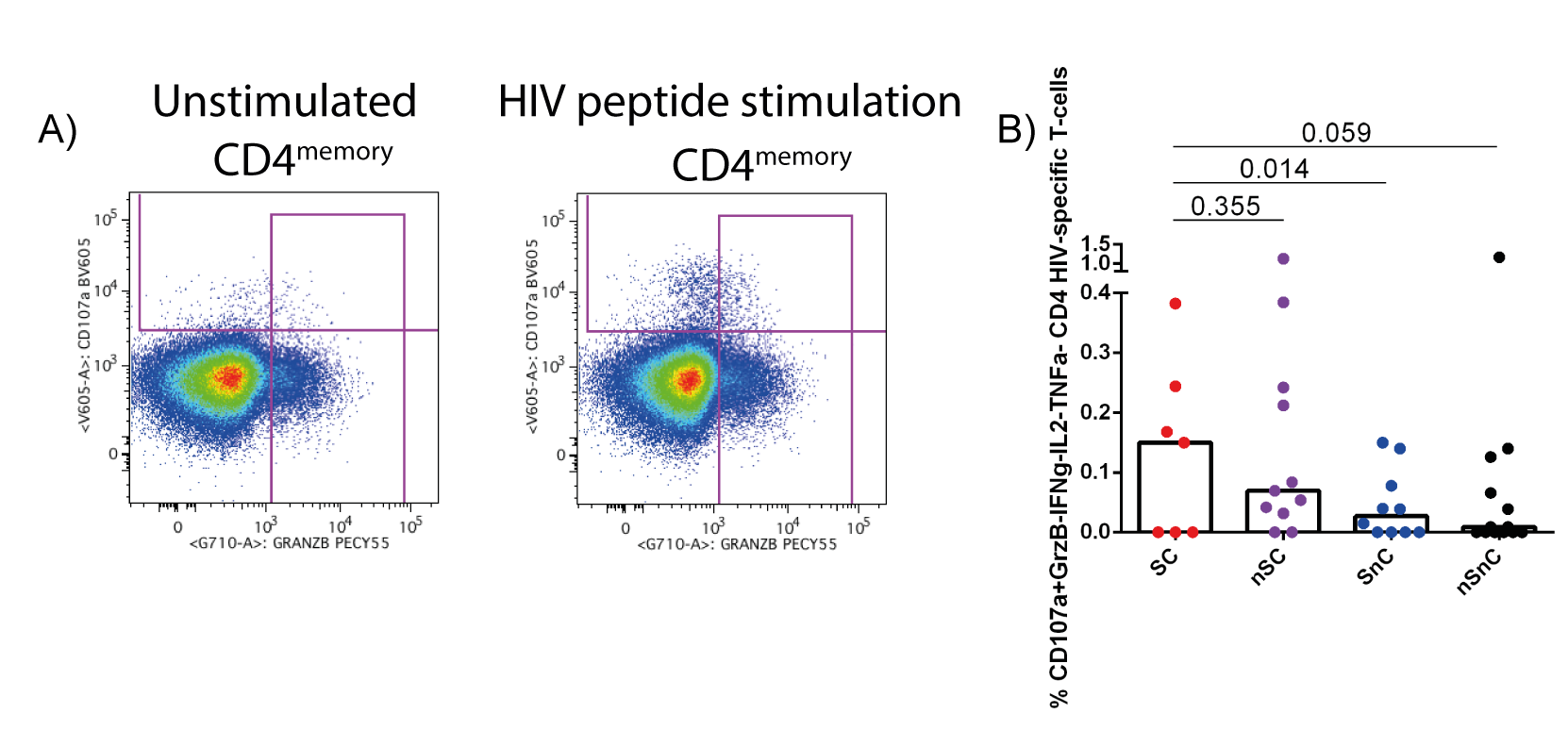

Supplement: Supplementary file 1 [file Image_1.TIF]

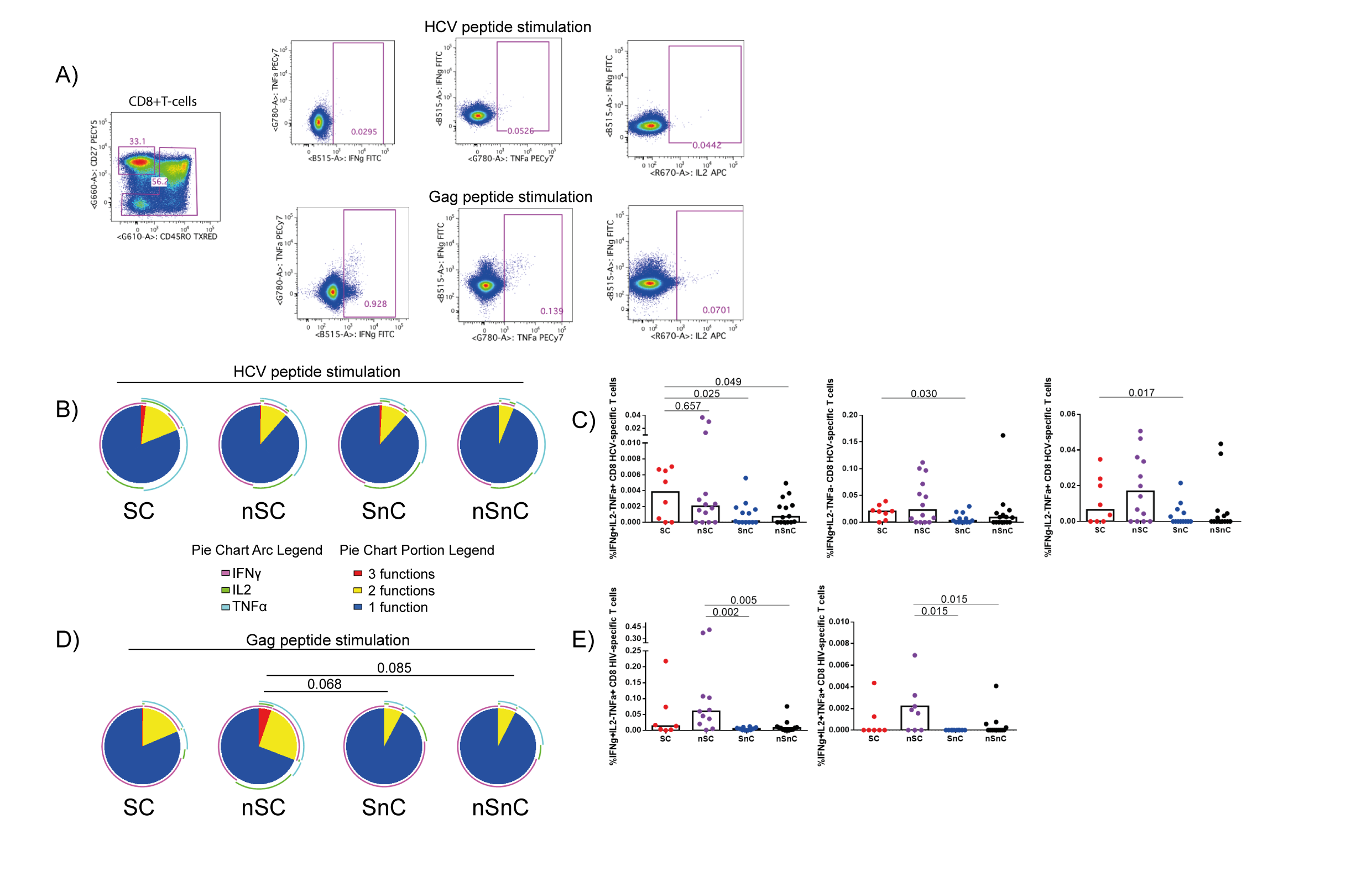

Supplement: Supplementary file 2 [file Image_2.TIF]

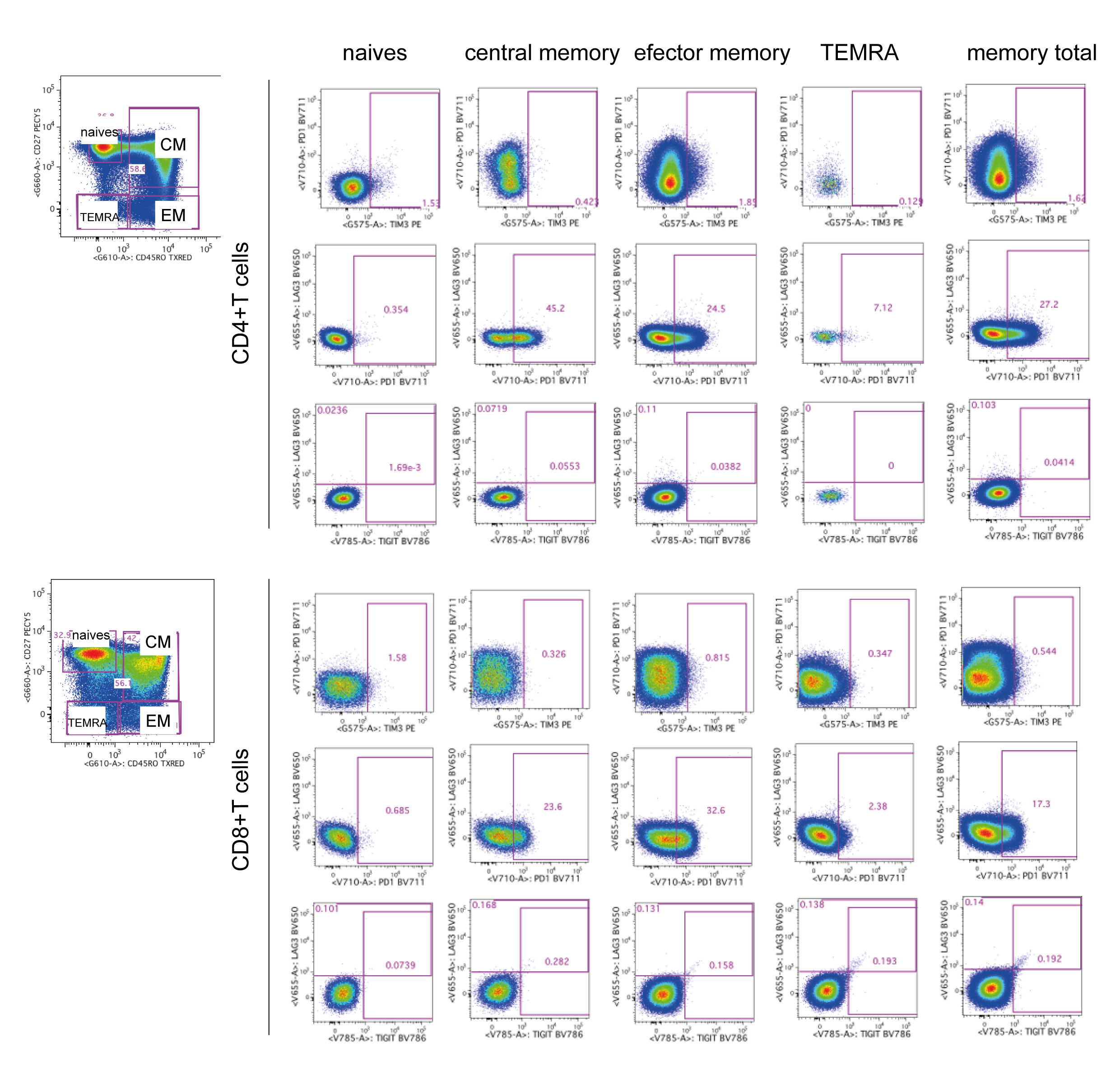

Supplement: Supplementary file 3 [file Image_3.TIF]

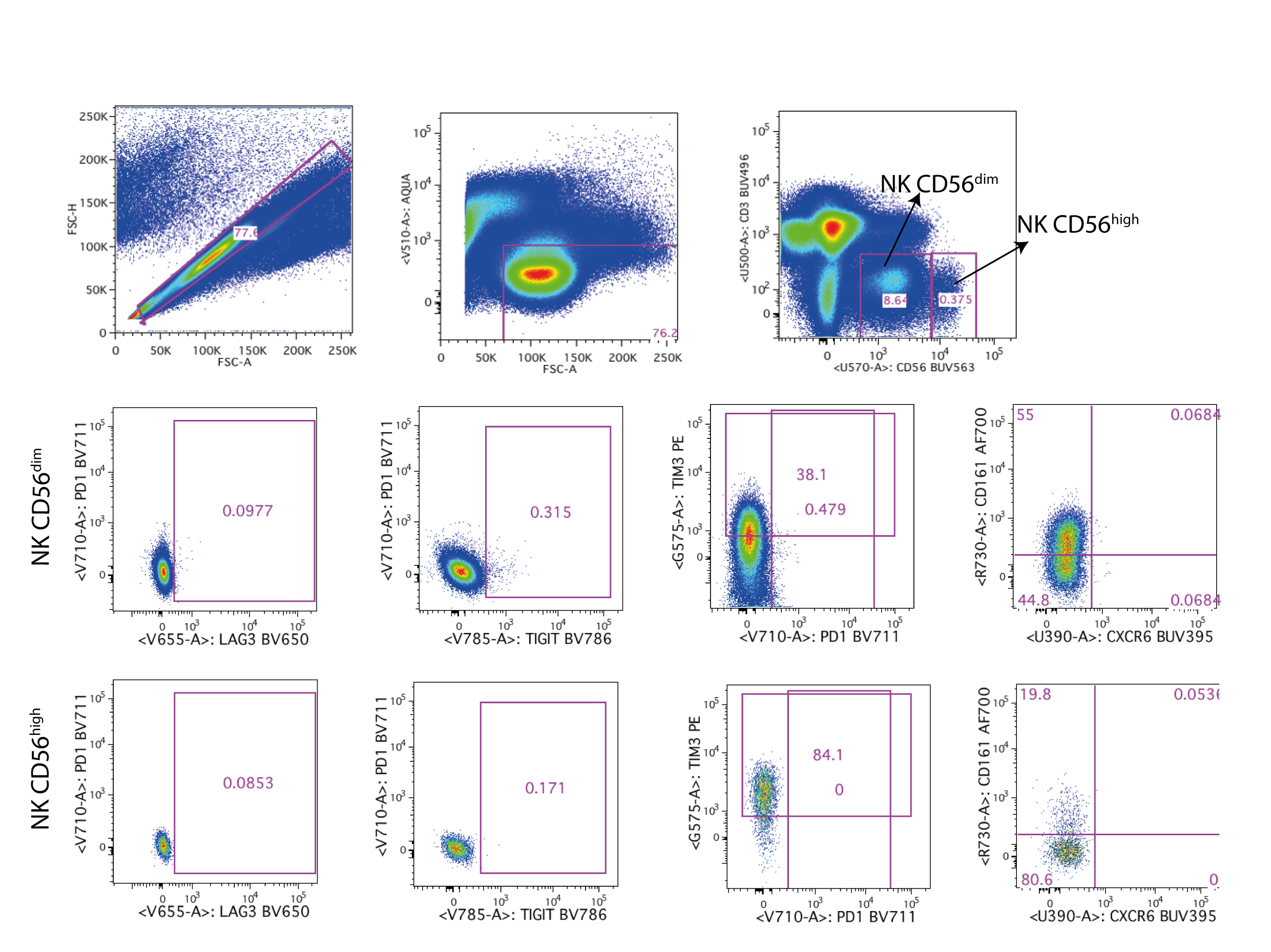

Supplement: Supplementary file 4 [file Image_4.TIF]
